# The Epigenetic Bivalency of Core Pancreatic β-Cell Transcription Factor Genes within Mouse Pluripotent Embryonic Stem Cells Is Not Affected by Knockdown of the Polycomb Repressive Complex 2, SUZ12

**DOI:** 10.1371/journal.pone.0097820

**Published:** 2014-05-20

**Authors:** Jennifer C. Y. Wong, Michelle M. Jack, Yan Li, Christopher O'Neill

**Affiliations:** 1 Centre for Developmental and Regenerative Medicine, Kolling Institute of Medical Research, Sydney Medical School, University of Sydney, Sydney, New South Wales, Australia; 2 Department of Endocrinology, Royal North Shore Hospital, Sydney, New South Wales, Australia; The Walter and Eliza Hall of Medical Research, Australia

## Abstract

This study assesses changes in activator and repressor modifications to histones associated with the core transcription factor genes most highly upregulated or downregulated in pancreatic β-cells relative to expression in an embryonic stem cell line. Epigenetic analysis of the *Oct4, Utf1, Nanog* and *Sox2* (pluripotency) and *Pdx1, Nkx6.1*, *Nkx2.2* and *MafA* (pancreatic β-cells) transcription factor genes in embryonic stem cells and a β-cell line (MIN6) showed the pluripotency genes were enriched for active (histone 3 trimethylated at lysine 4 and histone 3 acetylated at lysine 9) and depleted of repressor modifications (histone 3 trimethylated at lysine 27 and histone 3 trimethylated at lysine 9) around the transcription start site in mouse embryonic stem cells (D3), and this was reversed in MIN6 cells. The β-cell transcription factors were bivalently enriched for activating (histone 3 trimethylated at lysine 4) and repressor (histone 3 trimethylated at lysine 27) modifications in embryonic stem cells but were monovalent for the activator modification (histone 3 trimethylated at lysine 4) in the β-cells. The polycomb repressor complex 2 acts as a histone 3 lysine 27 methylase and an essential component of this complex, SUZ12, was enriched at the β-cell transcription factors in embryonic stem cells and was reduced MIN6. Knock-down of SUZ12 in embryonic stem cells, however, did not reduce the level of histone 3 trimethylated at lysine 27 at β-cell transcription factor loci or break the transcriptional repression of these genes in embryonic stem cells. This study shows the reduction in the total SUZ12 level was not a sufficient cause of the resolution of the epigenetic bivalency of β-cell transcription factors in embryonic stem cells.

## Introduction

There is a marked difference in the pattern of transcription from the genome between pluripotent cells (as exemplified by embryonic stem cells (ES cells) and each of the range of differentiated cell types that make up the body [1]–[3]. The pluripotent state requires the expression of a core set of transcription factors that include NANOG, POU5F1 (hereafter known as OCT4), UTF1 and SOX2 [4], [5]. Differentiation from the pluripotent state is accompanied by the repression of these core transcription factors and the active expression of different sets of transcription factors. The identity and timing of expression of new transcription factors defines the lineage formed during differentiation.

A range of covalent histone modifications within regulatory regions of genes are major determinants of gene expressivity [6], [7] and acetylation and methylation of specific lysine (K) residues on histone H3 have been the most extensively studied. Acetylation of H3K9 (H3K9ac) and tri-methylation of H3K4 (H3K4me3) are associated with an open, euchromatin structure that permits easier access of transcription factors and the activation of gene transcription [8]–[10]. Conversely, H3K27 and H3K9 tri-methylation (H3K27me3 and H3K9me3) generally serve as repressive chromatin modifications by the creation of a more closed conformation and these modifications are commonly associated with the formation of repressive heterochromatin [11], [12].

Genome-wide mapping of H3K4me3 and H3K27me3 in ES cells and differentiated cell-lineages demonstrate that genes which carry H3K4me3, but not H3K27me3, are generally actively expressed in ES cells. These include the core pluripotency transcription factor genes, *Nanog, Oct4, Sox2* and *Utf1*. Many genes were associated with both the active (H3K4me3) and repressive (H3K27me3) epigenetic signature. This bivalent state is thought to generate a condition whereby the gene is ‘poised’ for activation and held in a transcriptionally-ready state. Many of the genes possessing this bivalent domain are those that govern lineage specification. By contrast, genes marked by H3K27me3 but not H3K4me3 are stably repressed [13]–[15]. It is considered that the resolution of this bivalency allows the changes in expression required for lineage specific differentiation. The actions of the Polycomb group proteins (PcG) have a key role in this resolution. The Polycomb repressive complex (PRC) functions as a transcriptional repressor. PRC2 is responsible for catalyzing the repressive H3K27me3 modification [16], [17]. This complex is comprised of three main PcG proteins: the functional histone lysine methyltransferase, EZH2, and co-factors SUZ12 and EED [18], [19]. Each of these components is essential for PRC2 function.

An understanding of the processes that govern the formation of pancreatic β-cells is of importance since the loss or impaired function of pancreatic β-cells is a cause of diabetes. Functional β-cells have been derived from pluripotent ES cells and offer a potential therapy for diabetes. Yet, the efficiency and fidelity of these regenerative strategies remains low [20], [21]. A detailed understanding of the regulatory mechanisms governing the ontogeny of β-cells may progress therapeutic approaches. A range of transcription factors have been found to be associated with the formation and/or maintenance of the β-cell lineage, including PDX1, NKX6.1, NKX2.2, PAX4, MAFA and NEUROG3 [22], [23], but their epigenetic regulation has not been defined.

In this study, we systematically analyzed the differences in the epigenetic signatures (active modifications - H3K4me3 and H3K9ac; repressive modifications - H3K27me3 and H3K9me3) associated with a range of core transcription factor genes considered to encode the pluripotent state (*Nanog, Oct4, Utf1* and *Sox2*) and compared these with those considered necessary for differentiation to the β-cell lineage (*Pdx1, Nkx6.1, Nkx2.2, MafA* and *Pax4*). We show the putative β-cell lineage specifying genes exist in a bivalent state in pluripotent stem cells but this state was resolved to an active monovalent signature in a β-cell line. This occurred by the relative depletion of K3K27me3 in the β-cell line. Yet, surprisingly knock-down of H3K27 methylase activity (SUZ12) in stem cells did not reduce the level of H3K27me3 at β-cell loci, nor activate the expression of β-cell genes. This study suggests that the regulation of bivalency of histone modifications at core transcription factor genes with differentiation may require levels of regulation other than simply changes in the level of PcG activity.

## Materials and Methods

### Cell culture

Mouse ES cells from the D3 cell line [24] (P22–P28) (ATCC number CRL-1934) were maintained in Dulbecco's modified Eagle's medium with 4 mM L-glutamine, 4500 mg/L L-glucose and sodium pyruvate (DMEM) (Thermo Fisher Scientific, Waltham, MA), 10% (v/v) fetal bovine serum (FBS) (Invitrogen, Carlsbad, CA), 0.1 mM 2-mercaptoethanol (Sigma-Aldrich, St Louis, MO) and 1000 U/mL murine LIF (Millipore, Bedford, MA) on 0.1% (w/v) gelatin coated dishes. Mouse β-cell insulinoma line, MIN6 [25] (P23–P27) were cultured in DMEM with 15% (v/v) FBS and 0.1 mM 2-mercaptoethanol. Secretion of insulin in response to glucose challenge was confirmed (mouse insulin ELISA kit - Millipore). Both cell lines were cultured at 37°C at 5% CO_2_ in air.

### Microarray analysis using the Illumina Mouse WG-6 v2.0 Expression Beadchip

Total RNA was extracted from three independent samples of D3 and MIN6 cells with the Illustra RNAspin Mini RNA Isolation Kit (GE Healthcare, Little Chalfont, Buckinghamshire, UK). Microarrays were performed by the Australian Genome Research Facility (Sydney, NSW, Australia) using the Mouse WG-6 v2.0 Expression Beadchip (Illumina, San Diego, CA). Data output was normalized using the quantile normalization method in the Lumi Package in R statistical software [26] and analyzed in Partek Genomics package (Partek Inc, St Louis, MO). Student's *t*-test was used to identify genes with different expression in D3 and MIN6 samples (*p*<0.05). A further selection was then made for the genes that had a change in expression difference of at least 1.5 fold and *p*<0.05. Since the analysis only considered the major trend shifts in gene expression, multiple testing correction was not considered necessary. The subset of genes with different levels of expression between the two cell lines were categorized based on their functional assignment as assessed by Gene Ontology (GO) enrichment analysis [27] and the GO Classification Counter tool [28]. Sample genes from the microarray results were validated by quantitative RT-PCR (qRT-PCR) from aliquots of the RNA used for the microarray. The microarray data is accessible through NCBI Gene Expression Omnibus [29], GEO Series accession number GSE49077 (http://www.ncbi.nlm.nih.gov/geo/query/acc.cgi?acc=GSE49077).

### RT-PCR

Total RNA was reverse transcribed to cDNA in 5 mM MgCl_2_, 1X PCR Buffer II (50 mM KCl, 10 mM Tris-HCl, pH 8.3), 1 mM each of dATP, dCTP, dGTP and dTTP, 1 U RNase inhibitor, 2.5 µM random decamers, 2.5 U MuLV reverse transcriptase (Applied Biosystems, Foster City, CA) with 1 µg RNA per sample. RNA denatured at 65°C for 10 min prior to the addition of reverse transcriptase, and then incubated at 25°C for 10 min and 42°C for 30 min.

qRT-PCR in 25 µL reactions consisting of 1.5 mM MgCl_2_, 1X ImmoBuffer (2 mM Tris-HCl, 10 mM NaCl, 0.01 mM EDTA, 0.2 mM dithiothreitol (DTT), 5% glycerol, pH 7.5), 0.036 U IMMOLASE DNA polymerase (all from Bioline, London, UK), 0.08 mM each of dATP, dCTP, dGTP and dTTP (Applied Biosystems), 0.5 µM forward and reverse primers (sequences - Table S1), 0.13 µL 50X SYBR Green I nucleic acid gel stain (Invitrogen) and 16 ng cDNA per sample. Reactions in a Stratagene MX3000P (Stratagene, La Jolla, CA) using: one cycle of 95°C for 10 min and 40 cycles of 95°C for 20 s, 60°C for 30 s and 72°C for 30 s. Relative gene expression as described [30] was normalized against two housekeeping control genes, *Tbp* and *Actb*.

### DNA methylation analysis

Genomic DNA was extracted from D3 and MIN6 cells by Wizard Genomic DNA Purification Kit (Promega, Wisconsin, MN) and treated with the MethylEasy *Xceed* Rapid DNA Bisulfite Modification Kit (Human Genetics Signatures, Sydney, NSW, Australia). Nested bisulfite PCR in 25 µL total reaction consisting of 1.5 mM MgCl_2_, 1X GeneAmp PCR Gold Buffer (50 mM KCl, 15 mM Tris-HCl, pH 8.0), 0.2 mM each of dATP, dCTP, dGTP and dTTP, 1.25 U AmpliTaq Gold DNA polymerase (all from Applied Biosystems), 1 µM forward and reverse ‘outside’ or ‘inside’ primers (Table S2), and 2 µL bisulfite converted DNA per sample. Amplifying: 1 cycle at 95°C for 7 min, 10 cycles of 94°C for 1 min, 53°C for 1 min, 72°C for 1 min, followed by 25 cycles more cycles with extension for 30 s. Products from ‘outside’ primers were input DNA in a second round with ‘inside’ primers. Five deoxyadenosine overhangs were added to the 3′ end of each bisulfite PCR product in a 6 µL total reaction volume consisting of 3.3 mM MgCl_2_, 1X PCR Buffer (20 mM Tris-HCl, 50 mM KCl, pH 8.4), 0.165 mM each of dATP, dCTP, dGTP and dTTP, 3 U *Taq* DNA polymerase (all from Applied Biosystems) and 4 µL bisulfite PCR product. The reactions were incubated at 72°C for 12 min and immediately TA cloned into pCR4-TOPO vector and transformed with the TOP10-competent cells using the TOPO TA Cloning Kit for Sequencing (Invitrogen) following the manufacturer's instructions. Ten transformants were randomly selected and sequenced with *M13* forward 5′-GTAAAACGACGGCCAGTG primer. Sequencing reactions were performed by AGRF and analysis was carried out using the Sequence Scanner software version 1.0 (Applied Biosystems).

### Chromatin preparation

D3 or MIN6 cells (20×10^6^) were washed in 2 mL buffer (0.3M sucrose, 60 mM KCl, 15 mM NaCl, 5 mM MgCl_2_, 0.1 mM EGTA, 15 mM Tris-HCl (pH 7.5), 0.5 mM DTT, 0.1 mM PMSF and 3.6 ng/mL aprotinin, all from Sigma-Aldrich) on ice followed by lysis in the same buffer containing 0.4% (v/v) IGEPAL CA-630 (Sigma-Aldrich) for 10 min with agitation. Following centrifugation (3,200 g for 20 min at 4°C) over 1.2 M sucrose, the pellet was sheared using the ChIP-IT Express Enzymatic Kit (Active-Motif, Carslbad, CA) in preparation for chromatin immunoprecipitation (ChIP) assays.

### Quantitative ChIP

The specific ChIP-validated antibodies and non-immune control immunoglobulins used are shown in Table S3 and were used with the ChIP-IT Express Enzymatic Kit (Active-Motif) using 3.15 µg sheared DNA. The eluted immunoprecipitated DNA and a sample of ChIP input DNA were purified with the QIAquick PCR Purification Kit and then subjected to quantitative PCR (qPCR). qPCR was carried out as described for qRT-PCR except that 16 ng DNA was used instead of cDNA. Primers were designed to target a site within 1 kb of the transcription start site of the gene of interest (Table S4). Quantification of immunoprecipitated DNA was performed in relation to a standard curve that was created from a 10-fold dilution series of ChIP input DNA. Binding values from non-immune IgG were subtracted from the binding values of specific antibodies. The data is presented as the amount of DNA specifically bound (immunoprecipitated DNA) relative to the total amount of DNA (ChIP input DNA), expressed as a percentage.

### siRNA-mediated knockdown of Suz12 in D3 cells

siRNAs targeting *Suz12* (SUZ12 ON-TARGETplus SMARTpool siRNA), *Gapdh* (ON-TARGETplus GAPDH control pool) and a non-targeting control (ON-TARGETplus non-targeting pool) were obtained from Dharmacon (Thermo Fischer Scientific, Dharmacon division, Waltham, MA). We did not screen the effectiveness of the individual siRNAs that make up each of these pools. Transfections of siRNA into D3 cells were conducted using the DharmaFECT 1 transfection reagent following the manufacturer's instructions. The following transfection protocol was found to be necessary to achieve effective SUZ12 knock-down. Briefly, D3 cells were seeded at a density of 10,000 cells in 500 µL antibiotic free ES cell medium (“complete medium”) per well for 24 h. The cells were then transfected with 100 nM *Suz12*, *Gapdh* or non-targeting siRNA for 48 h, during which the transfection medium was removed after the first 3 h and replaced with complete medium. At 48 h transfection, the cells were harvested and replated at the original starting density. Twenty four h after replating, the cells were re-transfected as above for an additional 72 h. After 144 h (6 days) post-initial transfection, the cells were harvested and assessed for cell viability with 0.4% (w/v) trypan blue (Sigma-Aldrich), total cell number, immunofluorescence, gene expression by qRT-PCR and ChIP analysis.

### Immunofluorescence

All incubations were performed at room temperature unless specified otherwise. Transfected D3 cells on glass coverslips were fixed in ice cold 4% (w/v) paraformaldehyde (Sigma-Aldrich) for 30 min, followed by permeabilisation with 0.2% Triton X-100 (Sigma-Aldrich) for 30 min and blocking in 5% (v/v) goat serum (Sigma-Aldrich) for 1 h. Samples were incubated overnight at 4°C with rabbit SUZ12 antibody (Abcam) or rabbit IgG (Sigma-Aldrich) that was diluted 1∶250 in 5% goat serum. The cells were then incubated with FITC-labelled goat anti-rabbit IgG secondary antibody (Sigma-Aldrich) that was diluted 1∶200 in 5% goat serum for 1 h in the dark. DNA within the nuclei of the cells was counter-stained with 0.2 µg/mL propidium iodide (Sigma-Aldrich) for 15 min. Coverslips were mounted onto glass slides with VECTASHIELD mounting medium (Vector laboratories, Burlingame, CA). The slides were observed and optically sectioned with a Leica TCS SP5 II confocal microscope (Leica Microsystems, Wetzlar, Germany). Images were captured and processed using the Leica Application Suite Advanced Fluorescence software (Leica Microsystems). Analysis of SUZ12 expression was performed by measuring the intensity of fluorescence of at least 60 nuclei for each treatment condition. These nuclei were from at least six randomly selected colonies across three independent experiments of transfection and immunofluorescence. The intensity of fluorescence in single optical sections was measured by the Adobe Photoshop CS3 digital imaging software (Adobe Systems Inc., San Jose, CA).

### Statistical analysis

The IBM SPSS Statistics package version 19 (SPSS Inc., Chicago, IL) was used to perform all of the statistical analyses. A description of the statistical models used is provided in the legend to each figure or in text.

## Results

### Analysis of upregulated and downregulated genes between D3 and MIN6 cells

A total of 45,281 transcripts were examined in a whole mouse genome comparison between D3 and MIN6 cells. Approximately 21% (9,580 genes) of the transcripts had a level of expression that was at least 1.5-fold different between the two cell types; 4,604 genes had increased and 4,976 genes had decreased expression in MIN6 relative to D3 cells (*p*<0.05). To validate the results of the microarray analysis, six genes that were upregulated and six that were downregulated in MIN6 relative to D3 cells were analyzed by qRT-PCR. In all cases, the microarray and qRT-PCR results showed consistent trends of change in gene expression, although in all cases the qRT-PCR results showed a greater magnitude of change (**Table 1**). Those genes that showed different levels of expression (both upregulated and downregulated) in the two cell lines were categorized based on their functional assignment by GO enrichment analysis. Categorization according to the three parent GO domains found that of the genes upregulated in MIN6 relative to D3 cells, 71.3% were associated with biological processes, 7.4% with molecular function and 21.3% with cellular components. Of the genes upregulated in D3 relative to MIN6 cells, 89.1% were associated with biological processes, 6.7% with molecular function and 4.2% with cellular components. A more detailed functional assignment breakdown demonstrated that the genes upregulated in MIN6 compared to D3 cells were related to transport, metabolism, cell communication and response to external stimulus (Table S5).

**Table 1:** Validation of the microarray data by qRT-PCR.

	MIN6 (C_T_)^a^	D3 (C_T_)^a^	Microarray (expression fold change)	qRT-PCR (expression fold change)^b^
Upregulated transcripts in MIN6 relative to D3 cells				
* Ins2*	8.39±1.23	NE	121.63	N/A
* Ins1*	12.47±2.09	NE	99.59	N/A
* Pdx1*	18.42±1.32	NE	29.30	N/A
* Nkx6.1*	21.17±1.08	NE	27.10	N/A
* Pax4*	23.97±1.13	NE	3.17	N/A
* MafA*	25.43±2.14	NE	3.18	N/A
Downregulated transcripts in MIN6 relative to D3 cells				
* Nanog*	NE	22.38±1.25	−63.62	N/A
* Oct4*	NE	22.37±0.59	−63.46	N/A
* Sox2*	33.30±2.30	23.93±1.46	−11.01	−36.84±6.48
* Lin28*	26.67±2.39	19.78±1.15	−10.84	−44.99±4.26
* Dppa3*	30.24±1.21	25.68±1.23	−3.07	−10.58±1.30
* Dnmt3b*	25.81±1.18	21.37±0.12	−2.24	−7.25±0.38

Abbreviations: C_T_, threshold cycle; NE, not expressed; N/A, not applicable.

a Data presented as mean C_T_ ± standard deviation for N = 3 independent experiments. C_T_ values were normalised to the *Actb* and *Tbp* threshold cycles for each sample.

b Data presented as mean expression fold change in MIN6 relative to D3 cells ± standard deviation for N = 3 independent experiments. More information on the materials and methods used for this analysis is provided in the Materials and Methods S1 file.

The genes that were most upregulated in MIN6 relative to D3 cells were those required for insulin processing and secretion (*Ins2*, *Ins1*, *Cck*, *Scg3*, *Chgb*, *Isl1* and *Iapp*). Genes considered necessary for defining the β-cell phenotype (*Nkx2.2*, *Pdx1*, *Nkx6.1*, *MafA* and *Pax4*) also had increases expression in MIN6 cells. In D3 cells, the genes that were most upregulated relative to MIN6 cells were those related to pluripotency (*Dppa5, Dppa4, Nanog*, *Utf1*, *Oct4* and *Sox2*) (**Table 2**).

**Table 2:** Genes that were most upregulated in D3 and MIN6 cells as determined by microarray analysis.

Gene	Microarray (fold change)	p-value
**Upregulated transcripts in MIN6 relative to D3 cells**		
Insulin processing and secretion		
* Ins2*	121.63	p<0.001
* Ins1*	99.59	p<0.001
* Cck*	97.48	p<0.001
* Scg3*	96.15	p<0.001
* Chgb*	90.60	p<0.001
* Isl1*	82.95	p<0.001
* Iapp*	59.41	p<0.001
* Pcsk2*	8.25	p<0.001
* Egr1*	8.21	p<0.001
β-cell phenotype		
* Nkx2.2*	39.75	p<0.001
* Pdx1*	29.30	p<0.001
* Nkx6.1*	27.10	p<0.001
* MafA*	3.18	p<0.001
* Pax4*	3.17	p<0.001
**Upregulated transcripts in D3 relative to MIN6 cells**		
Pluripotency		
* Dppa5*	105.53	p<0.001
* Dppa4*	63.76	p<0.001
* Nanog*	63.63	p<0.001
* Oct4*	63.46	p<0.001
* Utf1*	21.99	p<0.001
* Sox2*	11.01	p<0.001

These changes in gene expression from the pluripotent state of D3 cells to the specific β-cell lineage are consistent with the expected outcomes. It is generally accepted that changes in the cell's epigenetic landscape accompany differentiation and are the basis for such changed patterns of gene expression. It was therefore of interest to assess the nature of changes in the expression of epigenetic modifiers in these two cell lines. The top 10 genes related to epigenetic processes that were most upregulated in D3 relative to MIN6 cells or MIN6 relative to D3 cells are shown (**Table 3**). As expected a large proportion of these genes were associated with histone modifications and chromatin remodeling, such as *Dnmt3b, Dnmt3l, Tcf3* and *Hmga1* (D3 cells), and *Smarca2*, *Hmgn3* and *Mll5* (MIN6 cells).

**Table 3:** The top 10 genes related to epigenetic processes that were most upregulated in D3 and MIN6 cells.

Gene	Microarray (fold change)	p-value	Function/physiological role
**D3 relative to MIN6**			
*Dnmt3l*	57.58	p<0.001	nuclear heterochromatin, methyltransferase activity, transcription repressor activity
*Gli2*	21.28	p<0.001	chromatin binding, promoter binding
*Dctd*	17.11	p<0.001	dCMP deaminase activity
*Dnmt3b*	15.01	p<0.001	DNA methyltransferase activity
*Tcf3*	11.53	p<0.001	histone H3 and H4 acetylation
*Hmga1*	11.33	p<0.001	regulation of cellular transcription, chromatin
*Lin28*	10.84	p<0.001	miRNA catabolic process, pre-miRNA processing, stem cell differentiation
*Myst4*	8.56	p<0.001	histone acetyltransferase activity,
*Mgmt*	8.15	p<0.001	methylated-DNA-(protein)-cysteine S-methyltransferase activity
*Zpf57*	6.35	p<0.001	DNA methylation during embryonic development, nuclear heterochromatin
**MIN6 relative to D3**			
*Smarca2*	25.10	p<0.001	chromatin organisation, nucleosome assembly
*Hmgn3*	19.87	p<0.001	chromatin binding
*Actl6b*	5.37	p<0.001	chromatin modification
*Srebf1*	5.00	p<0.001	positive regulation of histone deacetylation, chromatin binding
*Mll5*	4.86	p<0.001	chromation modification, DNA methyltransferase activity
*Snca*	4.64	p<0.001	histone binding
*Mbd1*	3.97	p<0.001	DNA methylation, heterochromatin
*Smarcc2*	3.91	p<0.001	chromatin modification and binding
*Hopx*	3.87	p<0.001	histone deacetylation
*Camta2*	3.76	p<0.001	chromatin binding, histone deacetylase binding

### The epigenetic status of *Actb*, *Oct4* and *Pdx1* in D3 and MIN6 cells

**Figure 1:**
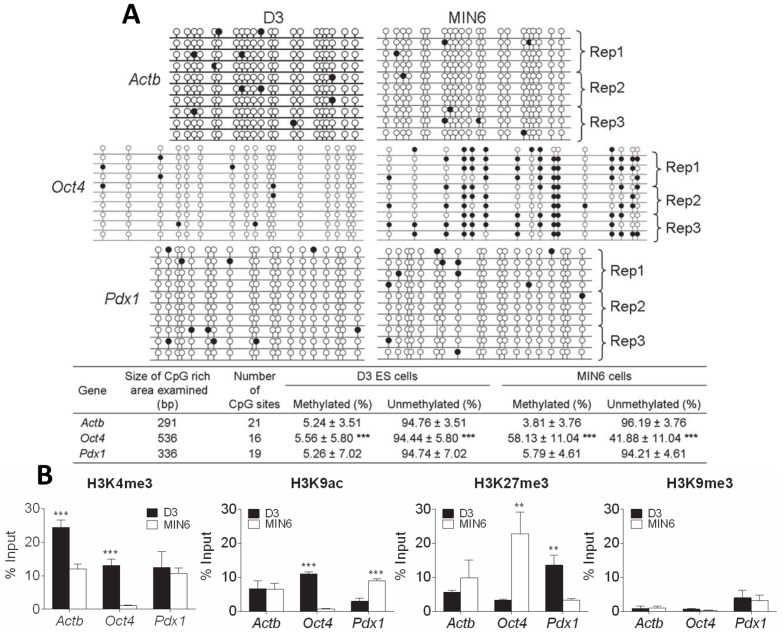
The epigenetic status of *Actb*, *Oct4* and *Pdx1* in D3 and MIN6 cells. **A**) The DNA methylation status of each CpG dinucleotide within a CpG-rich region of *Actb, Oct4* and *Pdx1* was determined by sequencing PCR clones of bisulfite converted DNA. Each line represents an individual clone and each circle an individual CpG. Open circles indicate an unmethylated CpG and closed circles a methylated CpG. The position of each circle is representative of the relative location along the length of the PCR product. Data is presented as the number of methylated or unmethylated CpGs relative to the total number of CpGs, expressed as a percentage. The results are the mean and standard deviation of 10 clones selected and sequenced across three independent experiments of cloning and transformations. **B**) ChIP assays for the active (H3K4me3 and H3K9me3) and repressive (H3K27me3 and H3K9me3) histone modifications were carried out on chromatin extracts from D3 (*black bars*) and MIN6 (*white bars*) cells. The presence of each histone modification at a locus within 1 kb of the transcription start site of *Actb*, *Oct4* and *Pdx1* was then quantified by qPCR analysis. Binding values of non-immune IgG were subtracted from the binding values of antibodies for each histone modification. The data is presented as the amount of DNA specifically bound relative to the total amount of DNA, expressed as a percentage. The results are the mean and standard deviation of three independent experiments. Statistically significant differences are denoted by **p<0.01 and ***p<0.001.

Two representative transcription factors of pluripotent and pancreatic β-cells (*Oct4* and *Pdx1*, respectively), together with the *Actb* housekeeping gene (control) were chosen for our initial analysis. The methylation state of each CpG dinucleotide within a CpG-rich region of *Actb*, *Oct4* and *Pdx1* was determined by sequencing PCR clones of bisulfite converted DNA. *Actb*, *Oct4* and *Pdx1* were primarily unmethylated in D3 cells (approximately 5% of CpG methylated). *Actb* and *Pdx1* remained unmethylated in MIN6 cells, but *Oct4* was relatively hypermethylated (56% of CpG methylated, *p*<0.001) (**Figure 1A**).

ChIP analysis of histone modifications within 1 kb of the transcription start site showed that the activating modifications (H3K4me3 and H3K9ac) were significantly enriched at *Oct4* in D3 relative to MIN6 cells (*p*<0.001). Conversely, the H3K27me3 (but not H3K9me3) repressive modification was enriched at *Oct4* in MIN6 relative to D3 cells (*p*<0.01). The *Pdx1* locus was associated with an increase in the activating H3K9ac (but not H3K4me3) modification in MIN6 compared to D3 cells (*p*<0.001). D3 cells had an enrichment of the repressive H3K27me3 modification at *Pdx1* (*p*<0.01). The *Actb* locus had similar levels of each of the modifications in both D3 and MIN6 cells, except for H3K4me3 which was higher in D3 cells (*p*<0.001). It was noteworthy that H3K9me3 was present at a lower level at all three loci in both cell types (**Figure 1B**).

Histone modifications around the transcription start site of *Pdx1* were bivalent in D3 cells, with enrichment of both the active (H3K4me3) and repressive (H3K27me3) histone modifications. This bivalency was not evident in the repressed *Oct4* loci in MIN6 cells. In MIN6 cells, the repressive H3K27me3 modification at *Pdx1* was reduced and both active histone modifications were enriched. This analysis shows a shift from the pluripotent state to the β-cell differentiated state was accompanied by the expected change from a bivalent epigenetic state to a monovalent active state and was unaffected by changes in DNA methylation. *Oct4* moved from an active to repressive signature for both histone and DNA modifications.

### The status of H3K4me3 and H3K27me3 at the key pluripotency and β-cell transcription factors in D3 and MIN6 cells

**Figure 2:**
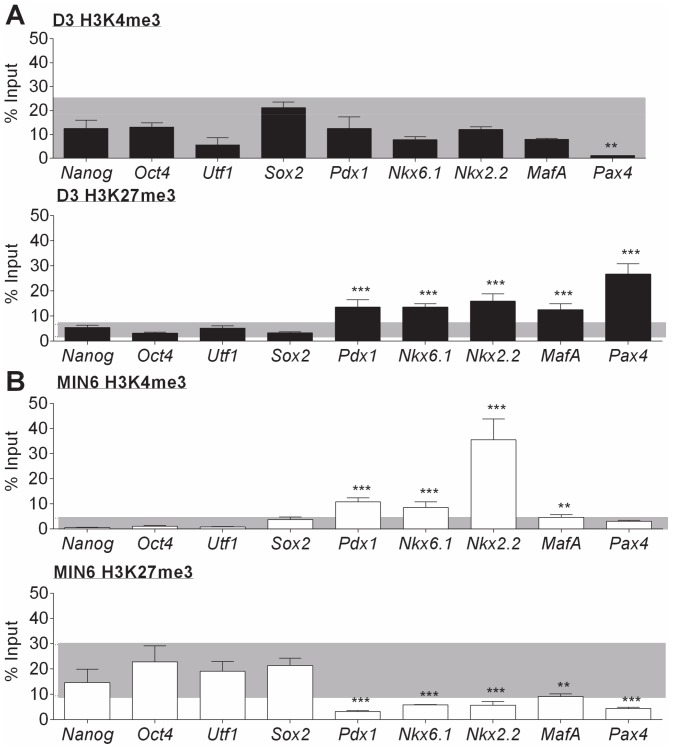
ChIP analysis for H3K4me3 and H3K27me3 at the key pluripotency and β-cell transcription factors in D3 and MIN6 cells. ChIP assays for the active H3K4me3 and repressive H3K27me3 histone modifications were carried out on chromatin extracts from **A**) D3 cells; and B) MIN6 cells. The presence of each histone modification at a locus within 1 kb of the transcription start site of the pluripotency (*Nanog, Oct4, Utf1* and *Sox2*) and β-cell (*Pdx1, Nkx6.1*, *Nkx2.2*, *MafA* and *Pax4*) transcription factors was then quantified by qPCR analysis. Binding values of non-immune IgG were subtracted from the binding values of antibodies for each histone modification. The data is presented as the amount of DNA specifically bound relative to the total amount of DNA, expressed as a percentage. As a benchmark to determine the level of histone binding that would indicate the significance of a histone modification at a gene of interest, the grey area represents the range two standard deviations around the mean of binding by the pluripotency genes (*Nanog*, *Oct4*, *Utf1* and *Sox2*) grouped together. The results are the mean and standard deviation of three independent experiments. Statistically significant differences in binding at a β-cell gene of interest in comparison to the benchmark range are denoted by **p<0.01 and ***p<0.001.

To assess whether bivalency of β-cell transcription factors was a general feature for such genes in D3 cells, we extended this analysis to a broader range of putative pluripotency and β-cell transcription factors. First, the consistency and robustness of the SUZ12 and JMJD3 ChIP assays in D3 and MIN6 cells was assessed (Figures S1 and S2). Using these validated assays, it was found that three of four other β-cell transcription factors (*Nkx6.1*, *Nkx2.2* and *MafA*) were bivalent in D3 cells (**Figure 2A**) but were enriched for only the active H3K4me3 modification in MIN6 cells (**Figure 2B**). By contrast, the *Pax4* locus was enriched with only the repressive H3K27me3 in D3 cells and had low levels of both H3K4me3 and H3K27me3 in MIN6 cells (**Figure 2**). Three pluripotency transcription factors (*Utf1*, *Nanog* and *Sox2*) displayed a similar pattern of histone modifications as *Oct4* in both D3 and MIN6 cells (**Figure 2**). Thus, a shift from a bivalent to an active epigenetic signature appears to be a feature of these lineage-associated transcription factors. This result may implicate the net level of H3K27me3 at loci as an important determinant of changes in gene expression with differentiation. We therefore next assessed the presence of the H3K27 methylase (as exemplified by its critical component SUZ12) and the demethylase (JMJD3) at these loci.

### The status of SUZ12 and JMJD3 binding at the key β-cell transcription factors in D3 and MIN6 cells

**Figure 3:**
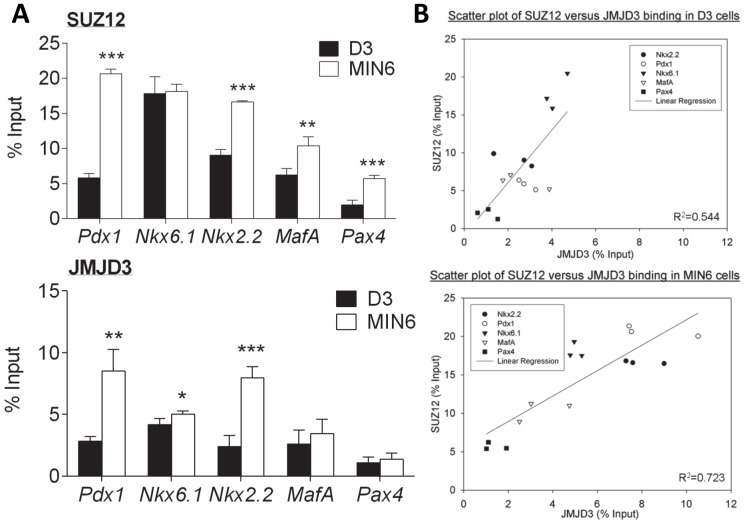
Analysis of SUZ12 and JMJD3 binding at the key β-cell transcription factors in D3 and MIN6 cells. **A**) ChIP assays for SUZ12 and JMJD3 were carried out on chromatin extracts from D3 (*black bars*) and MIN6 (*white bars*) cells. The presence of SUZ12 and JMJD3 at a locus within 1 kb of the transcription start site of the β-cell transcription factors, *Pdx1*, *Nkx6.1*, *Nkx2.2*, *MafA* and *Pax4* was then quantified by qPCR analysis. Binding values of non-immune IgG were subtracted from the binding values of SUZ12 and JMJD3 antibodies. The data is presented as the amount of DNA specifically bound relative to the total amount of DNA, expressed as a percentage. The results are the mean and standard deviation of three independent experiments. Statistically significant differences in SUZ12 and JMJD3 binding between D3 and MIN6 cells are denoted by *p<0.05, **p<0.01 and ***p<0.001. **B**) Scatter plot representation of SUZ12 versus JMJD3 binding at the key β-cell transcription factors in D3 and MIN6 cells. The results of three independent experiments are shown. A linear regression was performed on all of the data in each plot.

The H3K27 methylase component (SUZ12) was significantly enriched at the *Pdx1* (*p*<0.001), *Nkx2.2* (*p*<0.001), *MafA* (*p*<0.01) and *Pax4* (*p*<0.001) loci in MIN6 compared to D3 cells, but *Nkx6.1* showed similar high levels of enrichment of SUZ12 in both cell lines. The H3K27me3 demethylase (JMJD3) was also enriched at the *Pdx1* (*p*<0.01), *Nkx6.1* (*p*<0.05) and *Nkx2.2* (*p*<0.001) loci in MIN6 compared to D3 cells, but there was no difference in the levels of enrichment at the *MafA* or *Pax4* loci between the two cell types (**Figure 3A**). Linear regression analysis showed a positive relationship between SUZ12 and JMJD3 binding at the β-cell loci in both D3 (R^2^ = 0.544) and MIN6 cells (R^2^ = 0.723) (**Figure 3B**). The similar relationship between SUZ12 and JMJD3 binding at the β-cell transcription factor loci in both D3 and MIN6 cells was a surprising finding given the marked differences in the relative levels of H3K27me3 at these loci in the two cell types. It is possible that the kinetic properties of these enzymes can result in large changes in net methylation levels with only small changes in the amount of enzyme present. To assess this possibility, we next assessed the effect of modulating SUZ12 levels on methylation levels.

### Analysis of siRNA-mediated knockdown of Suz12 in D3 cells

siRNA-mediated knockdown of *Suz12* in D3 cells was performed and the effect on gene expression assessed. Preliminary analysis showed that the SUZ12 protein was highly stable in D3 cells. After 72 h transfection, *Suz12* expression was 73±6% lower in the *Suz12* siRNA sample relative to the scrambled siRNA treatment (*p*<0.001) but there was no difference in SUZ12 protein expression between treatments (*p*>0.05) (data not shown). To achieve effective knockdown of SUZ12 in D3 cells, it was necessary to transfect D3 cells with *Suz12* siRNA for at least 144 h.

**Figure 4:**
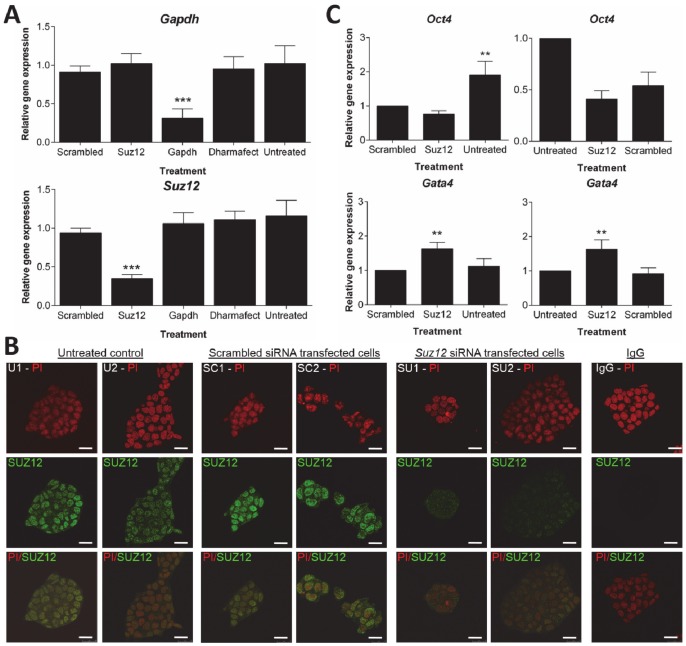
Analysis of siRNA-mediated knockdown of *Suz12* in D3 cells after 144 h transfection. D3 cells were transfected with 100*Gapdh*, *Suz12* or non-targeting ‘scrambled’ siRNA using the DharmaFECT 1 transfection reagent in antibiotic-free ES cell medium. Untreated control cells were maintained in antibiotic-free ES cell medium only. The ‘Dharmafect’ control was cultured in transfection medium without siRNA. **A**) qRT-PCR analysis was performed to test the expression status of *Gapdh* and *Suz12* at 144 h transfection. The data is expressed as the average relative gene expression ± standard deviation in comparison to the scrambled siRNA. Quantified values were normalized against two housekeeping genes, *Tbp* and *Actb.* The results are the mean and standard deviation of three independent experiments. Statistically significant differences in gene expression levels are denoted by ***p<0.001. **B**) Representative immunostaining images of the untreated control (U1-2), scrambled siRNA (SC1-2), *Suz12* siRNA (SU1-2) and cell colonies stained with non-immune IgG (IgG) are shown. DNA within the cell nuclei were counter-stained red with propidium iodide (PI) (*top panel*). Cells that expressed SUZ12 were stained green with anti-SUZ12 antibody (*middle panel*). Images from these two channels were merged to show co-localisation (*bottom panel*). Images were single-equatorial confocal sections (0.7 µm) of cell colonies. The images shown here are representative of four independent replicate experiments with at least three colonies per replicate examined. The scale bars are 25 µm. **C**) qRT-PCR analysis of *Oct4* and *Gata4* expression at 144 h transfection. The data is expressed as the average relative gene expression ± standard deviation in comparison to the scrambled siRNA (left column) or untreated sample (right column). Quantified values were normalized against two housekeeping genes, *Tbp* and *Actb.* The results are the mean and standard deviation of three independent experiments. Statistically significant differences in gene expression levels are denoted by **p<0.01.

The *Suz12* or *Gapdh* (positive control) siRNA treatments caused significantly lower levels of target gene expression compared to the scrambled siRNA control treatment at 144 h transfection (65±5% and 69±12% lower expression after the *Suz12* or *Gapdh* siRNA, respectively, *p*<0.001) (**Figure 4A**). Immunofluorescence localization of SUZ12 showed that *Suz12* siRNA treatment caused a significant reduction in the levels of nuclear SUZ12 staining (71±6% lower expression in the sample relative to the scrambled siRNA treatment, *p*<0.001) (**Figure 4B**).

In D3 cells with SUZ12 knockdown, expression of the five β-cell genes (*Pdx1*, *Nkx6.1*, *Nkx2.2*, *MafA* and *Pax4*) remained undetectable by qRT-PCR. This indicates that this treatment failed to initiate the transcription of these lineage defining genes in D3 cells. The *Suz12*-siRNA treatment also had no effect on the expression of *Oct4* relative to scrambled control, although the transfection process itself had some adverse affects on *Oct4* expression (*p*<0.01). A marker of ES cell differentiation, *Gata4*, did show a significantly higher level of expression in *Suz12* siRNA treated cells (*p*<0.01) (**Figure 4C**) but not after transfection of control siRNA, indicating that a reduction in PcG did have some effect on the differentiation status of D3 cells, but not on the genes of interest.

**Figure 5:**
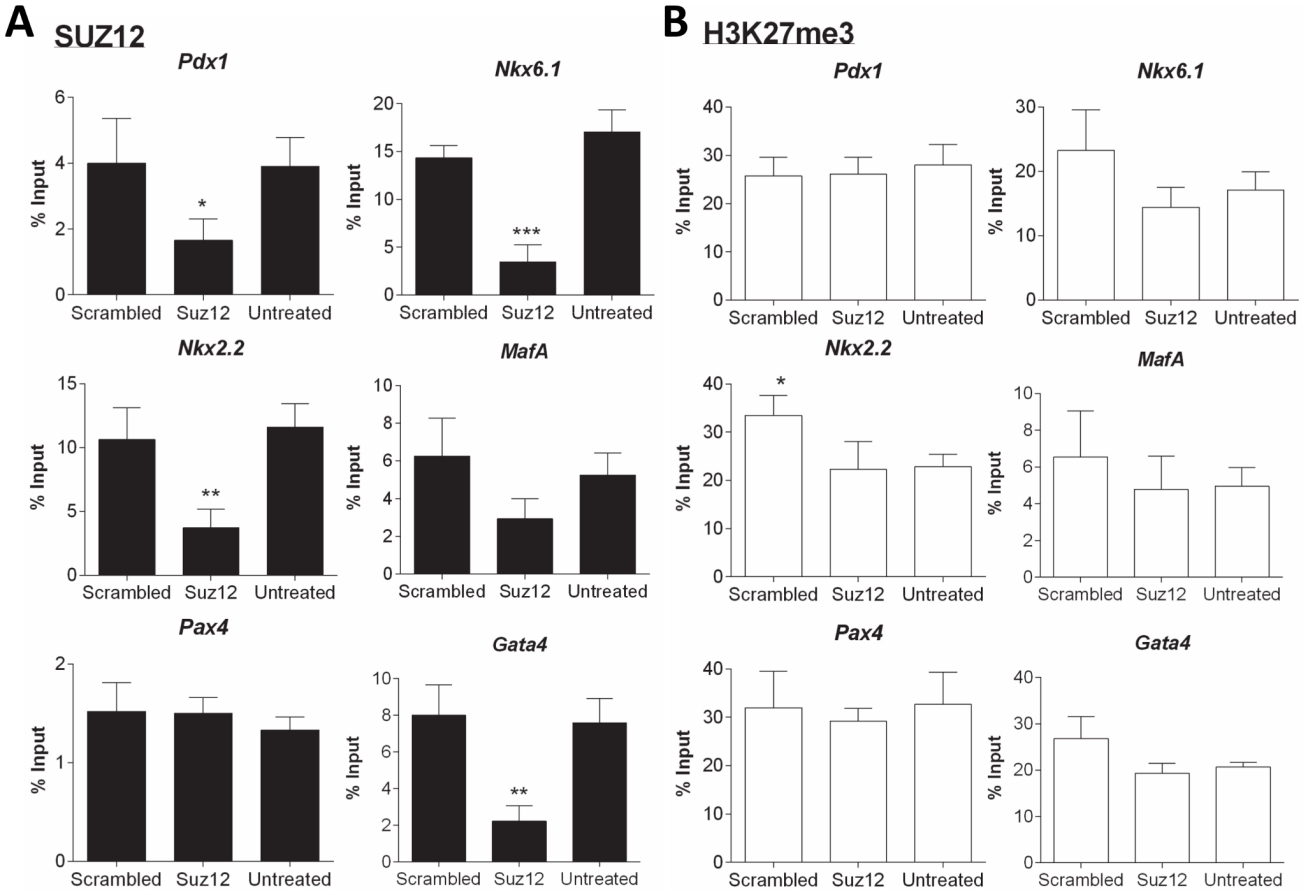
The effect of siRNA-mediated transfection on SUZ12 and H3K27me3 binding at *Gata4* and the key β-cell transcription factors in D3 cells. D3 cells were transfected with 100*uz12* or non-targeting ‘scrambled’ siRNA using the DharmaFECT 1 transfection reagent in antibiotic-free ES cell medium. Untreated control cells were maintained in antibiotic-free ES cell medium only. ChIP assays for **A**) SUZ12 and **B**) H3K27me3 were carried out on chromatin extracts from samples after 144 h transfection. The presence of SUZ12 and H3K27me3 at a locus within 1 kb of the transcription start site of *Gata4* and the β-cell transcription factors, *Pdx1*, *Nkx6.1*, *Nkx2.2*, *MafA* and *Pax4* were then quantified by qPCR analysis. Binding values of non-immune IgG were subtracted from the binding values of SUZ12 or H3K27me3 antibody. The data is presented as the amount of DNA specifically bound relative to the total amount of DNA, expressed as a percentage. The results are the mean and standard deviation of three independent experiments. Statistically significant differences in SUZ12 or H3K27me3 binding at a loci are denoted by *p<0.05, **p<0.01 and ***p<0.001.

*Suz12* siRNA treatment caused a decrease in SUZ12 binding at several of the β-cell loci (*Pdx1*, *p*<0.05; *Nkx2.2*, *p*<0.01; and *Nkx.1*, *p*<0.001). This was also the case for the marker gene for ES cell differentiation, *Gata4* (*p*<0.01). Two β-cell transcription factors (*Pax4* and *MafA*) showed no significant change in SUZ12 enrichment (p>0.05) (**Figure 5A**). The marked reduction in the levels of SUZ12 at *Pdx1, Nkx2.2* and *Nkx6.1* loci was not accompanied by a decrease in the level of H3K27me3 enrichment at these loci. This was also the case for the differentiation marker *Gata4*. (**Figure 5B**). Thus, a marked change in the level of SUZ12 present at loci had no detectable effect on the level of H3K27me3 at these loci. The result shows that the level of PRC2 at loci of pluripotent cells is not of itself a primary or sufficient determinant of maintaining the epigenetic bivalency of β-cell lineage-specific genes in an epigenetically poised state. It is not known whether this is a general feature of all bivalent loci, nor was the status of constitutively expressed genes (such as the housekeeping genes) assessed.

## Discussion

This study shows that a range of core transcription factors that define the pluripotent state are characterized by an active epigenetic signature in ES cells and have a characteristic repressive epigenetic signature in the MIN6 β-cell insulinoma line. By contrast, a range of putative core β-cell transcription factor genes displayed an epigenetically bivalent signature in ES cells, and this was generally resolved to an active monovalent signature in MIN6 β-cells. This epigenetic resolution resulted from the relative depletion of H3K27me3 from the transcription start site at these loci in the β-cell line. These changes in the epigenetic signatures were accompanied by the expected changes in the relative levels of expression of each of these transcription factor genes between the two lineages.

Methylation of H3K27 is most commonly catalyzed by the PRC2 complex, composed of EZH2, SUZ12 and EED [18], [19]. PRC2 has a role in maintaining the pluripotent state and has markedly reduced expression upon the differentiation of ES cells [31]–[35]. Conversely, the H3K27me3 demethylases, UTX and JMJD3 are reported to be absent from bivalent genes in ES cells and are recruited to some of these genes, including the homeotic Hox gene family, upon differentiation [36], [37]. This is consistent with our observation that the differentiation marker *Gata4* was upregulated upon knock-down of SUZ12 in ES cells. Yet, this upregulation of *Gata4* occurred without any detectable decrease in the amount of H3K27me3 associated with this loci. Furthermore, the knockdown of SUZ12 (or the treatment with the known PRC2 inhibitor, 5 µM 3-Deazaneplanocin A, data not shown) did not cause a change in the level of H3K27me3 at each of the β-cell transcription factor loci in ES cells or a change in their level of expression. Surprisingly, SUZ12 was present at high levels at the β-cell transcription factor loci in both ES cells and β-cells despite there being a large relative reduction of H3K27me3 at these loci in β-cells. While SUZ12 was reduced in MIN6 cells relative to the same site in ES cells this was accompanied by a similar reduction in the amount of JMJD3 (a H3K27me3 demethylase) at the transcription start site of most of these loci. In both cell types, there was a direct positive relationship between the levels of SUZ12 (methylase) and JMJD3 (demethylase). This relationship was surprising and different from that expected based upon the observations that the homeotic HOX family genes showed UTX and JMJD3 dependent loss of H3K27me3 upon differentiation of ES cells [36], [37]. It is possible that resolution of epigenetic bivalency of homeotic genes and lineage-specifying genes involve different epigenetic mechanisms and this possibility requires further investigation.

This unexpected relationship between PRC2 and JMJD3 might suggest that some regulatory mechanisms exist between these two enzymes that govern total H3K27me3 levels. It was therefore expected that the net level of H3K27me3 would result from an equilibrium between the actions of methylases and demethylases, and that reduction in methylase activity by the knock-down of SUZ12 would change the equilibrium in favour of lower H3K27me3. Yet this was not found to be the case. Genetic studies show that SUZ12 is essential for PRC2 activity [38], [39] so the markedly reduced levels of SUZ12 detected after these treatments might have been expected to result in a reduction in the PRC activity. Perhaps some unexpected compensatory changes in demethylase activity occurred to maintain high net levels of H3K27me3 at these loci. It is noteworthy that SUZ12 was highly refractory to siRNA-mediated knockdown, requiring treatment over 144 h to achieve a transient reduction of ∼70%. This suggests that SUZ12 is a highly stable protein. The study design does not exclude the possibility that the remaining protein was sufficient to maintain normal levels of methylation, yet a finding that the PRC inhibitor, Deazaneplanocin A, was also without effect may suggest otherwise. If the remaining protein was sufficient to maintain the H3K27me3, this may indicate that ES cells normally possess a very large ‘reserve capacity’ of this enzyme activity. The long and extensive transfection procedure required to achieve efficient knockdown did cause some changed gene expression and some impact of this on the results cannot be entirely excluded. While MIN6 β-cells are a transformed cell line, they share many of the characteristics of normal β-cells. For example, cultured MIN6 cells are homogeneous in morphology and grow in clusters that contain neuroendocrine granules similar to *in vivo* β-cells [25], [40]. MIN6 cells also express liver-type glucose transporter that *in vivo* β-cells require for glucose sensing [41]. Importantly, MIN6 cells have the ability for glucose-stimulated insulin secretion at a rate similar to normal mouse islets [42], Despite these similarities, the possibility that their transformed state impacts on their epigenetic profile should be born in mind.

This study provides the first comparative analysis of some aspects of the epigenetic state of the core transcription factor genes that code for the pluripotent and pancreatic β-cell lineages. The results identified an association of both histone modifications and DNA methylation at the key pluripotency and β-cell transcription factors in D3 and MIN6 cells. The findings show that loss of H3K27me3 at the key β-cell transcription factors is associated with β-cell lineage specification. A novel association between SUZ12 and JMJD3 at a regulatory region of the β-cell loci in D3 and MIN6 cells was identified, which inferred a role for an equilibrium between H3K27 methylase and demethylase activity in the epigenetic regulation of these genes. However, inhibition or depletion of cellular levels of PRC2-associated SUZ12 protein did not reduce H3K27me3 binding at the β-cell loci or result in the expression of β-cell genes in treated D3 cells. The results indicate that multiple levels of control of the maintenance of the bivalent epigenetic state of core developmental genes occurs within ES cells.

## Supporting Information

Figure S1 **ChIP analysis across three antibody concentrations for SUZ12.** ChIP assays using a range of SUZ12 antibody concentrations were carried out on chromatin extracts from A) D3 mouse ES cells (*black bars*) and B) MIN6 pancreatic β-cells (*white bars*). The presence of SUZ12 at a locus within 1 kb of the transcription start site of *Brachyury* was then quantified by qPCR. Binding values of non-immune IgG were subtracted from the binding values of SUZ12 antibody. The data is presented as the amount of DNA specifically bound relative to the total amount of DNA, expressed as a percentage. The results are the mean and standard deviation of three independent experiments. The results of an analysis of variance are stated in the text. Information on the materials and methods used for this analysis is provided in the Materials and Methods S1 file.
(DOCX)

Figure S2 **ChIP analysis across three antibody concentrations for JMJD3.** ChIP assays using a range of JMJD3 antibody concentrations were carried out on chromatin extracts from A) D3 mouse ES cells (*black bars*) and B) MIN6 pancreatic β-cells (*white bars*). The presence of JMJD3 at a locus within 1 kb of the transcription start site of *Nodal* was then quantified by qPCR. Binding values of non-immune IgG were subtracted from the binding values of JMJD3 antibody. The data is presented as the amount of DNA specifically bound relative to the total amount of DNA, expressed as a percentage. The results of an analysis of variance are stated in the text. The results are the mean and standard deviation of three independent experiments. The results of an analysis of variance are stated in the text. Information on the materials and methods used for this analysis is provided in the Materials and Methods S1 file.
(DOCX)

Table S1 **Validation of the microarry data by qRT-PCR.**
(PDF)

Table S2 **Quantitative RT-PCR primer sequences.**
(PDF)

Table S3 **Bisulfite PCR primer sequences.**
(PDF)

Table S4 **Primary antibodies and immunoglobulins used in ChIP.**
(PDF)

Table S5 **Quantitative PCR primer sequences of gene promoters.**
(PDF)

Table S6 **Gene Ontology enrichment analysis of genes upregulated in one cell line relative to the other.**
(PDF)

Materials and Methods S1 (DOCX)
