# Supplementary figures and images for: The Epigenetic Bivalency of Core Pancreatic β-Cell Transcription Factor Genes within Mouse Pluripotent Embryonic Stem Cells Is Not Affected by Knockdown of the Polycomb Repressive Complex 2, SUZ12
Source: PLoS One. 2014 May 20;9(5):e97820. doi: 10.1371/journal.pone.0097820 (PMC4028244; doi:10.1371/journal.pone.0097820)

Figure S1


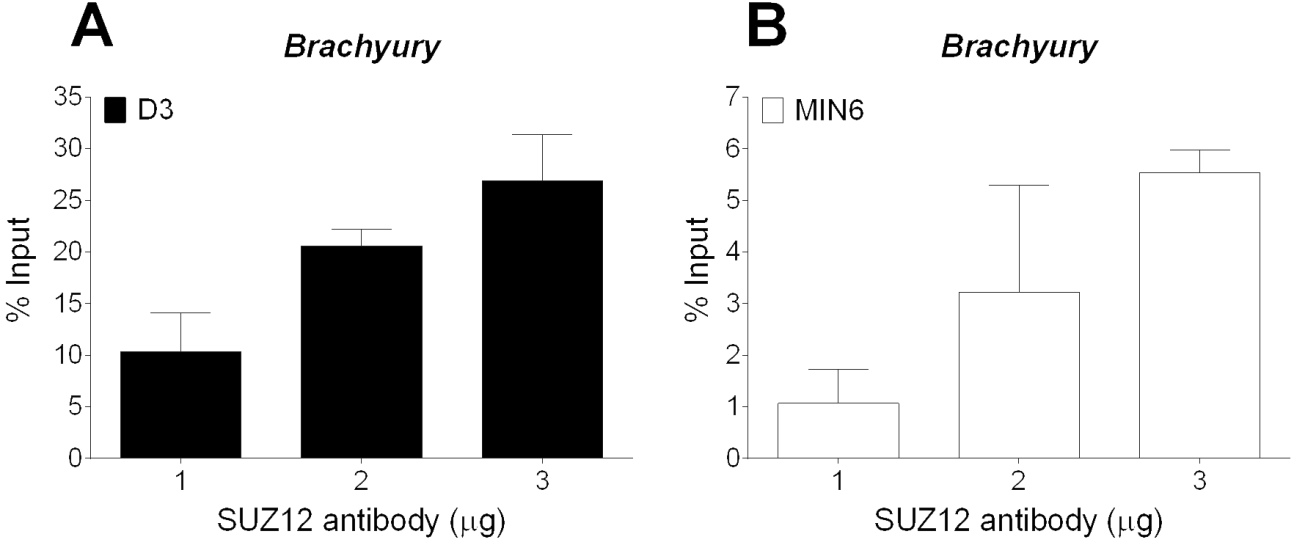

Supplement: Figure S1 — ChIP analysis across three antibody concentrations for SUZ12. ChIP assays using a range of SUZ12 antibody concentrations were carried out on chromatin extracts from A) D3 mouse ES cells (black bars) and B) MIN6 pancreatic β-cells (white bars). The presence of SUZ12 at a locus within 1 kb of the transcription start site of Brachyury was then quantified by qPCR. Binding values of non-immune IgG were subtracted from the binding values of SUZ12 antibody. The data is presented as the amount of DNA specifically bound relative to the total amount of DNA, expressed as a percentage. The results are the mean and standard deviation of three independent experiments. The results of an analysis of variance are stated in the text. Information on the materials and methods used for this analysis is provided in the Materials and Methods S1 file. (DOCX) [file pone.0097820.s001.docx]

**Figure S2.**


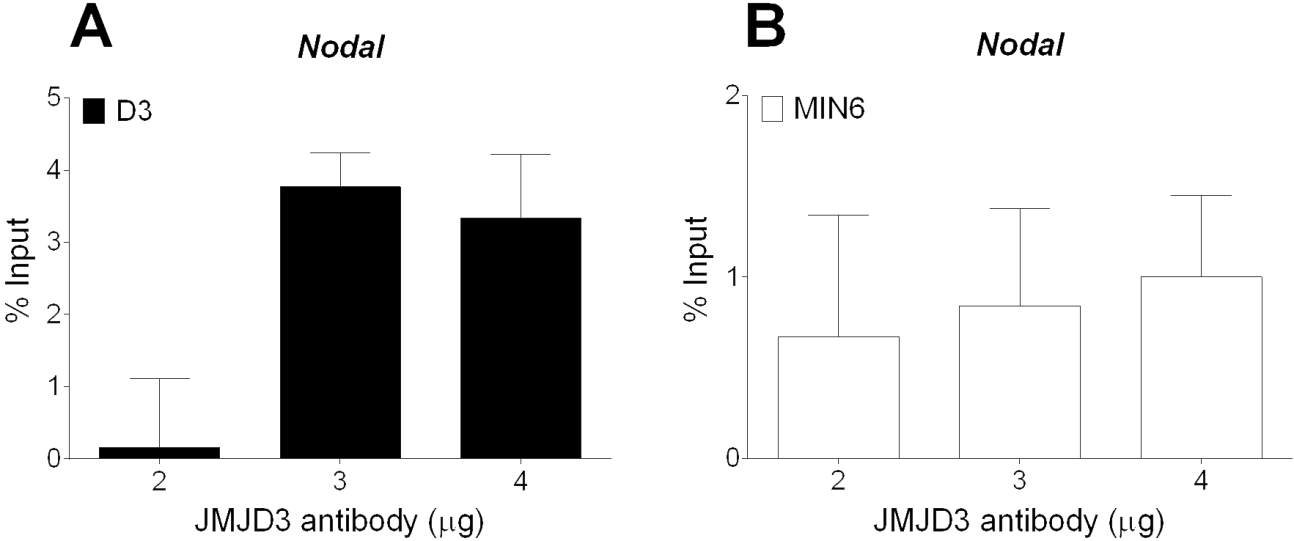

Supplement: Figure S2 — ChIP analysis across three antibody concentrations for JMJD3. ChIP assays using a range of JMJD3 antibody concentrations were carried out on chromatin extracts from A) D3 mouse ES cells (black bars) and B) MIN6 pancreatic β-cells (white bars). The presence of JMJD3 at a locus within 1 kb of the transcription start site of Nodal was then quantified by qPCR. Binding values of non-immune IgG were subtracted from the binding values of JMJD3 antibody. The data is presented as the amount of DNA specifically bound relative to the total amount of DNA, expressed as a percentage. The results of an analysis of variance are stated in the text. The results are the mean and standard deviation of three independent experiments. The results of an analysis of variance are stated in the text. Information on the materials and methods used for this analysis is provided in the Materials and Methods S1 file. (DOCX) [file pone.0097820.s002.docx]
