# Supplementary material for: The Epigenetic Bivalency of Core Pancreatic β-Cell Transcription Factor Genes within Mouse Pluripotent Embryonic Stem Cells Is Not Affected by Knockdown of the Polycomb Repressive Complex 2, SUZ12
Source: PLoS One. 2014 May 20;9(5):e97820. doi: 10.1371/journal.pone.0097820 (PMC4028244; doi:10.1371/journal.pone.0097820)
Supplement: Table S1 — Validation of the microarry data by qRT-PCR. (PDF) [file pone.0097820.s003.pdf]

Table S1. Validation of the microarry data by qRT-PCR

	MIN6 (C_T)[a]	D3 (C_T)[a]	Microarray (expression fold change)	qRT-PCR (expression fold change)[b]
Upregulated transcripts in MIN6 relative to D3 cells				
Ins2	8.39 ± 1.23	NE	121.63	N/A
Ins1	12.47 ± 2.09	NE	99.59	N/A
Pdx1	18.42 ± 1.32	NE	29.30	N/A
Nkx6.1	21.17 ± 1.08	NE	27.10	N/A
Pax4	23.97 ± 1.13	NE	3.17	N/A
MafA	25.43 ± 2.14	NE	3.18	N/A
Downregulated transcripts in MIN6 relative to D3 cells				
Nanog	NE	22.38 ± 1.25	-63.62	N/A
Oct4	NE	22.37 ± 0.59	-63.46	N/A
Sox2	33.30 ± 2.30	23.93 ± 1.46	-11.01	-36.84 ± 6.48
Lin28	26.67 ± 2.39	19.78 ± 1.15	-10.84	-44.99 ± 4.26
Dppa3	30.24 ± 1.21	25.68 ± 1.23	-3.07	-10.58 ± 1.30
Dnmt3b	25.81 ± 1.18	21.37 ± 0.12	-2.24	-7.25 ± 0.38

Abbreviations: C_T, threshold cycle; NE, not expressed; N/A, not applicable.

[a]Data presented as mean C_T ± standard deviation for N = 3 independent experiments. C_T values were normalised to the *Actb* and *Tbp* threshold cycles for each sample

[b]Data presented as mean expression fold change in MIN6 relative to D3 cells ± standard deviation for N = 3 independent experiments.
