# Supplementary material for: The Epigenetic Bivalency of Core Pancreatic β-Cell Transcription Factor Genes within Mouse Pluripotent Embryonic Stem Cells Is Not Affected by Knockdown of the Polycomb Repressive Complex 2, SUZ12
Source: PLoS One. 2014 May 20;9(5):e97820. doi: 10.1371/journal.pone.0097820 (PMC4028244; doi:10.1371/journal.pone.0097820)
Supplement: Table S2 — Quantitative RT-PCR primer sequences. (PDF) [file pone.0097820.s004.pdf]

**Table S2. Quantitative RT-PCR primer sequences**

| Gene | Forward 5'-3' | Reverse 5'-3' | Product size | Gene Accession No. |
|---|---|---|---|---|
| *Actb* | CTAAGGCCAACCGTGAAAAG | GTACGACCAGAGGCATACG | 109 | NM_007393 |
| *Cyp4x1* | ACCACAATGTGCATCAAGGA | TGTCCATCTGGGAGGGTAAG | 95 | NM_001003947 |
| *Dnmt3b* | AGTGACCAGTCCTCAGACACGAAG | ATCAGAGCCATTCCCATCATCTAC | 210 | NM_001003961.3 |
| *Dppa3* | AAGGGTCCGCACTTTGTTGT | CATCTGAATGGCTCACTGTCC | 129 | NM_025274 |
| *Gapdh* | TGCCCCCATGTTTGTGATG | TGTGGTCATGAGCCCTTCC | 151 | NM_008084.2 |
| *Gata4* | CACAAGATGAACGGCATCAACC | CAGCGTGGTGGTGGTAGTCTG | 111 | NM_008092 |
| *Hoxb1* | CATCAGCCTACGACCTCCTC | CGCTGTCTTAGGTGGGTTTC | 119 | NM_008266 |
| *Ins1* | CCAGCTATAATCAGAGACCA | GTGTAGAAGAAGCCACGCT | 197 | NM_008386 |
| *Ins2* | CCCTGCTGGCCCTGCTCTT | AGGTCTGAAGGTCACCTGCT | 213 | NM_001185083 |
| *Lin28* | GTCTTTGTGCACCAGAGCAA | CTTTGGATCTTCGCTTCTGC | 193 | NM_145833 |
| *MafA* | GAGGAGGTCATCCGACTGAA | CTCTGGAGCTGGCACTTCTC | 125 | NM_194350 |
| *Pax4* | TGGCTTCCTGTCCTTCTGTGAGG | TCCAAGACACCTGTGCGGTAGTAG | 242 | NM_011038 |
| *Pdx1* | ACCGCGTCCAGCTCCCTTTC | CAACATCACTGCCAGCTCCACC | 212 | NM_008814 |
| *Nanog* | AGGGTCTGCTACTGAGATGCTCTG | CAACCACTGGTTTTTCTGCCACCG | 361 | NM_028016 |
| *Nkx2.2* | CCGGGCGGAGAAAGGTATG | CTGTAGGCGGAAAAGGGGA | 156 | NM_010919 |
| *Nkx6.1* | ACTTGGCAGGACCAGAGAGA | CGATTTGTGCTTTTTCAGCA | 266 | NM_144955 |
| *Nr2f1* | TGAGAAGCCAGTACCCCAAC | GCTCGATGACAGAGGAGGAC | 93 | NM_010151 |
| *Oct4* | GAGGGATGGCATACTGTGGAC | GGTGTACCCCAAGGTGATCC | 272 | NM_013633 |
| *Sox2* | CCCCTTTTATTTTCCGTAGTTGTAT | TCAAACTGTGCATAATGGAGTAAAA | 151 | NM_011443 |
| *Suz12* | CTTCCAGCCAGAAGAAAACG | GCATGGCCACTTCATATTCC | 121 | NM_199196.2 |
| *Tbp* | GAATTGTACCGCAGCTTCAAAA | AGTGCAATGGTCTTTAGGTCAAGTT | 76 | NM_013684 |
| *Tcfap2b* | CCACGTCAACGATCCCTACT | CTTCTGAGCCCACTTCTTGG | 92 | NM_009334 |
| *Trpc5* | CCAAAACAAATGAGGGGCTA | TGGAGAGGCTTCTTCTTGGA | 123 | NM_009428 |
