# Supplementary material for: The Epigenetic Bivalency of Core Pancreatic β-Cell Transcription Factor Genes within Mouse Pluripotent Embryonic Stem Cells Is Not Affected by Knockdown of the Polycomb Repressive Complex 2, SUZ12
Source: PLoS One. 2014 May 20;9(5):e97820. doi: 10.1371/journal.pone.0097820 (PMC4028244; doi:10.1371/journal.pone.0097820)
Supplement: Table S3 — Bisulfite PCR primer sequences. (PDF) [file pone.0097820.s005.pdf]

Table S3. Bisulfite PCR primer sequences

Gene	Forward 5'-3'	Reverse 5'-3'	Distance from transcription start site
Actb			
Outer	ATRGGTTTGGATAAAGATTTAGAGGTT	TAACTAATACCTCACTACAAAATCCTAAAA	+487 to +108
Inner	TTTTAATATTGTGTATTTTTAAGATGGATT	TAACTAATACCTCACTACAAAATCCTAAAA	+398 to +108
Oct4			
Outer	AGGTGTAATGGTTGTTTTGTTTTGGTTTTG	TAACCCATCACCCCCACCTAATAAAAATAA	-125 to +482
Inner	TATGGGTTGAAATATTGGGTTTATTTATAT	TCTAAAACCAAATATCCAACCATAA	-85 to +450
Pdx1			
Outer	TTARGTGGGGTGTTTTAGAGTTTATGGT	AAACTTCCCTACTCCAATAATCCCAA	+86 to +464
Inner	TTTTTAGTTAGTTTTTGGTTTTTTAGGAGAGT	AAACTTCCCTACTCCAATAATCCCAA	+129 to +464
