# Supplementary material for: The Epigenetic Bivalency of Core Pancreatic β-Cell Transcription Factor Genes within Mouse Pluripotent Embryonic Stem Cells Is Not Affected by Knockdown of the Polycomb Repressive Complex 2, SUZ12
Source: PLoS One. 2014 May 20;9(5):e97820. doi: 10.1371/journal.pone.0097820 (PMC4028244; doi:10.1371/journal.pone.0097820)
Supplement: Table S4 — Primary antibodies and immunoglobulins used in ChIP. (PDF) [file pone.0097820.s006.pdf]

Table S4. Primary antibodies and immunoglobulins used in ChIP

Antibody	Supplier	Catalogue No.	Concentration of antibody
IgG from mouse serum	Sigma-Aldrich	I5381	As per antibody of interest
IgG from rabbit serum	Sigma-Aldrich	I5006	As per antibody of interest
Mouse monyclonal to H3K27me3	Active-Motif	39535	2.5 µg
Rabbit polyclonal to H3K4me3	Active-Motif	39159	2.5 µg
Rabbit polyclonal to H3K9Ac	Abcam	AB4441	2.5 µg
Rabbit polyclonal to H3K9Me3	Abcam	AB8898	2.5 µg
Rabbit polyclonal to JMJD3	Abcam	AB85392	4 µg
Rabbit polyclonal to SUZ12	Abcam	AB12073	3 µg
