# Supplementary material for: The Epigenetic Bivalency of Core Pancreatic β-Cell Transcription Factor Genes within Mouse Pluripotent Embryonic Stem Cells Is Not Affected by Knockdown of the Polycomb Repressive Complex 2, SUZ12
Source: PLoS One. 2014 May 20;9(5):e97820. doi: 10.1371/journal.pone.0097820 (PMC4028244; doi:10.1371/journal.pone.0097820)
Supplement: Table S5 — Quantitative PCR primer sequences of gene promoters. (PDF) [file pone.0097820.s007.pdf]

Table S5. Quantitative PCR primer sequences of gene promoters

Gene	Forward 5'-3'	Reverse 5'-3'	Distance from transcription start site
Actb	GCAGGCCTAGTAACCGAGACA	AGTTTTGGCGATGGGTGCT	-335
Cyp4x1	GTGGACCAGATGAAGCCATT	TGACTTCTCTGTCCCCATCC	-560
Gata4	CACAAGATGAACGGCATCAACC	CAGCGTGGTGGTGGTAGTCTG	-404
Hoxb1	CAGTCTCCCTCTCCCTTCCT	CTTATCCCAGAACCCCCATT	-595
MafA	GGATCTGAGACACCGAAGGA	GTGGCAGATTCTGAGGTTCC	-759
Pax4	GGCCCATGCTTGAGTACATT	ACCTTGTGGCTCTACCCTGA	-240
Pdx1	TTGGGAGCGGTTTTGTAAAG	CTCTCTCAGTCGCCTGATCC	-268
Nanog	TTAAAAAGCCGCACTTTTGG	GACCTTGCTGCCAAAGTCTC	-421
Nkx2.2	GAGGACCAAGCCTCTGAACA	TCCTTGGAGGGCTTAAGATG	-486
Nkx6.1	GCTATTGGAGGCGGTGTTTA	GGGGCCAAAATGAGAACTTT	-660
Nr2f1	AGGGGAAAAGGACTTTCCAA	AATCAAAGGCGACTGACTGG	-119
Oct4	GGCTCTCCAGAGGATGGCTGAG	TCGGATGCCCCATCGCA	-361
Sox2	CCATCCACCCTTATGTATCCAAG	CGAAGGAAGTGGGTAAACAGCAC	-361
Tcfap2b	TTAGTGGCTGCATTGATTCG	TGGGTCACAGAATCCACAGA	-228
Trpc5	GGGTTCTCCACAGCTCTCAG	CCACCCCTAGATCCCAAAAT	-490
Utf1	TGCCCTCTTCACTCGATCTT	ACTCAGGACCTTCGGTGAGA	-709
