# Supplementary material for: The Epigenetic Bivalency of Core Pancreatic β-Cell Transcription Factor Genes within Mouse Pluripotent Embryonic Stem Cells Is Not Affected by Knockdown of the Polycomb Repressive Complex 2, SUZ12
Source: PLoS One. 2014 May 20;9(5):e97820. doi: 10.1371/journal.pone.0097820 (PMC4028244; doi:10.1371/journal.pone.0097820)
Supplement: Table S6 — Gene Ontology enrichment analysis of genes upregulated in one cell line relative to the other. (PDF) [file pone.0097820.s008.pdf]

Table S6. Gene Ontology enrichment analysis of genes upregulated in one cell line relative to the other

Gene Ontology category	Proportion of differentially expressed genes (%)
Genes upregulated in D3 relative to MIN6 cells	
Metabolism	14.2
Development	12.0
Cell differentiation	10.1
Biosynthesis	5.4
Morphogenesis	5.4
Binding	4.7
Transport	4.7
Cell communication	4.1
Embryonic development	4.1
Signal transduction	3.5
Nucleobase, nucleoside, nucleotide and nucleic acid metabolism	3.5
Regulation of gene expression, epigenetic	2.2
Other*	26.4
Genes upregulated in MIN6 relative to D3 cells	
Transport	13.7
Metabolism	11.2
Cell	8.7
Cell communication	6.8
Response to external stimulus	5.6
Behavior	5.0
Binding	5.0
Protein binding	3.7
Cell-cell signaling	3.7
Protein metabolism	3.7
Receptor binding	3.1
Carbohydrate metabolism	3.1
Enzyme regulator activity	3.1
Cytoplasm	2.5
Signal transduction	2.5
Cell differentiation	2.5
Intracellular	2.5
Other*	13.7

* Consists of categories that contained less than 2% of the differentially expressed genes (29 categories for D3 and 12 for MIN6)
