# Supplementary material for: The Epigenetic Bivalency of Core Pancreatic β-Cell Transcription Factor Genes within Mouse Pluripotent Embryonic Stem Cells Is Not Affected by Knockdown of the Polycomb Repressive Complex 2, SUZ12
Source: PLoS One. 2014 May 20;9(5):e97820. doi: 10.1371/journal.pone.0097820 (PMC4028244; doi:10.1371/journal.pone.0097820)
Supplement: Materials and Methods S1 — (DOCX) [file pone.0097820.s009.docx]

Supplementary Materials and Methods

Supplementary information for Table 1

The microarray results were validated by qRT-PCR analysis, whereby the relative expression level of 12 genes by D3 and MIN6 cells was quantified. These genes were selected from the microarray data and spanned between those with low relative gene expression to those with high relative gene expression between D3 and MIN6 cells.

qRT-PCR analysis of six chosen genes that were upregulated in MIN6 relative to D3 cells (*Ins2, Ins1, Pdx1, Nkx6.1, Pax4* and *MafA*) showed that they were not expressed at a detectable level in D3. As such, the qRT-PCR expression fold change between D3 and MIN6 cells could not be calculated. However, expression of these genes was detected in MIN6 cells by qRT-PCR. Analysis of the C_T_ values found that the trend in expression of these six genes by MIN6 cells was consistent with the microarray expression fold change levels. That is, a low C_T_ value of 8.39 ± 1.23 for *Ins2* correlated with a high microarray fold change of 121.63, whereas a higher C_T_ value of 25.43 ± 2.14 for *MafA* correlated with a low microarray fold change of 3.18.

qRT-PCR analysis of six chosen genes that were down-regulated in MIN6 relative to D3 cells (*Nanog, Oct4, Sox2, Lin28, Dppa3* and *Dnmt3b*) gave an expression change level of a higher magnitude than that of the microarray results. The trend in relative gene expression, however, remained consistent; the genes that had a relatively low or high fold change in the microarray data also had a relatively low or high fold change in the qRT-PCR data. Therefore, the qRT‑PCR results confirmed and properly reflected the trends in the microarray to show that the microarray results were valid.

Supplementary information for Figures S1 and S2

ChIP assays were carried out with three concentrations of SUZ12 and JMJD3 antibodies in D3 and MIN6 cells. This was to assess the optimal concentration of antibodies for use. *Brachyury* and *Nodal* were chosen as the positive gene controls based on the finding by Dahle *et al*. (2010) that they were bound by SUZ12 and JMJD3, respectively, in ES cells.

*SUZ12 antibody*

SUZ12 binding at the *Brachyury* locus increased in an antibody concentration dependent manner in both D3 (p < 0.001) (Supplementary Figure 1A) and MIN6 cells (p < 0.01) (Supplementary Figure 1B). The level of binding between the use of 2 µg or 3 µg antibody, however, was not different from each other (p > 0.05). This indicated that at least 2 µg SUZ12 antibody was sufficient to saturate the antigen at the *Brachyury* locus in both cell lines.

*JMJD3 antibody*

There was also a significant effect of antibody concentration on JMJD3 binding at the *Nodal* locus in D3 cells (p < 0.01). However, there was no difference in the level of binding between the use of 3 µg or 4 µg antibody (p > 0.05) (Supplementary Figure 2A). This implied that saturation of the antigen was reached with 3 µg JMJD3 antibody in D3 cells.

In MIN6 cells, there was no difference in the level of JMJD3 binding at the *Nodal* locus between the three tested concentrations of antibody (p > 0.05) (Supplementary Figure 2B). This suggested that 2 µg JMJD3 antibody was adequate for binding to be at equilibrium in MIN6 cells. It was thus decided that ChIP assays on D3 and MIN6 cells would be carried out using 3 µg SUZ12 and 4 µg JMJD3 antibodies.

**Reference:**

Dahle O, Kumar A & Kuehn MR. (2010). Nodal signaling recruits the histone demethylase Jmjd3 to counteract polycomb-mediated repression at target genes. Sci Signal 3(127), ra48.
